# Diffusion coefficients and dissociation constants of enhanced green fluorescent protein binding to free standing membranes

**DOI:** 10.1016/j.dib.2015.10.002

**Published:** 2015-10-26

**Authors:** Franziska A. Thomas, Ilaria Visco, Zdeněk Petrášek, Fabian Heinemann, Petra Schwille

**Affiliations:** a Max Planck Institute of Biochemistry, Am Klopferspitz 18, D-82152 Martinsried, Germany; b Graz University of Technology, Institute of Biotechnology and Biochemical Engineering, Petersgasse 10-12/I, A-8010 Graz, Austria; c Roche Diagnostics, Nonnenwald 2, D-82377 Penzberg, Germany

## Abstract

Recently, a new and versatile assay to determine the partitioning coefficient $K_{P}$ as a measure for the affinity of peripheral membrane proteins for lipid bilayers was presented in the research article entitled, “Introducing a fluorescence-based standard to quantify protein partitioning into membranes” [1]. Here, the well-characterized binding of hexahistidine-tag (His_6_) to NTA(Ni) was utilized. Complementarily, this data article reports the average diffusion coefficient D of His_6_-tagged enhanced green fluorescent protein (eGFP-His_6_) and the fluorescent lipid analog ATTO‐647N‐DOPE in giant unilamellar vesicles (GUVs) containing different amounts of NTA(Ni) lipids. In addition, dissociation constants $K_{d}$ of the NTA(Ni)/eGFP-His_6_ system are reported. Further, a conversion between $K_{d}$ and $K_{P}$ is provided.

**Specifications table**

**Table:** 

Subject area	*Biophysics*
More specific subject area	*Molecular Biophysics*
Type of data	*Table, figure*
How data was acquired	*Fluorescence Correlation Spectroscopy, Confocal Microscopy using a LSM 780 with a ConfoCor 3 unit (Zeiss, Jena, Germany)*
Data format	*Analyzed*
Experimental factors	*GUVs consisting of DOPC and 2, 3, 4 or 5 mol% DGS-NTA(Ni), labeled with 0.05 mol% ATTO-647N-DOPE*
Experimental features	*Titration of eGFP-His*_*6*_*to* the *GUVs*
Data source location	*Max Planck Institute of Biochemistry, Martinsried, Germany*
Data accessibility	The data are provided within this article

**Value of the data**

• **We provide the first valuable characterization of the eGFP-His_6_/NTA(Ni) system with precise dissociation constants *K_d_* for increasing percentages of DGS-NTA(Ni) in the membrane.**
• **The eGFP-His_6_/NTA(Ni) dissociation constants could serve as reference for other His_6_-tagged proteins reconstituted in GUVs.**
• **We provide a conversion between *K_d_* and *K*_P_ for the His_6_-NTA(Ni) system, which can be extended to any protein-lipid interaction with a known 1:1 stoichiometry.**
• **Protein diffusion coefficients could be used as an indicator of crowding effects.**
• **As for DOPC/DGS-NTA(Ni) the lipid dynamics is independent of increasing protein concentrations, the ATTO-647N-DOPE diffusion coefficient could serve as a standard.**

## 1 Data

Hexahistidine-tag (His_6_) binding to Nickel (Ni) chelated with nitrilotriacetic acid (NTA) is a well-characterized process [2], [3] and it is extensively used to reconstitute protein systems in giant unilamellar vesicles (GUVs) [4], [5], [6]. We made GUVs consisting of 1,2-di-(9*Z*-octadecenoyl)-*sn*-glycero-3-phosphocholin (DOPC) and 2, 3, 4 or 5 mol% 1,2-di-(9*Z*-octadecenoyl)-*sn*-glycero-3-[(*N*-(5-amino-1-carboxypentyl)iminodiacetic acid)succinyl] nickel salt (DGS-NTA(Ni)), labeled with 0.05 mol% ATTO-647N-DOPE. These GUVs were incubated with increasing amounts of His_6_-tagged enhanced green fluorescent protein (eGFP-His_6_) and point fluorescence correlation spectroscopy (FCS) was performed both at the top pole of the GUVs and in solution. From the obtained FCS auto-correlation functions the diffusion coefficient D of both eGFP-His_6_ and ATTO-647N-DOPE as well as the dissociation constant $K_{d}$ of the NTA(Ni)/eGFP-His_6_ system were calculated.

## 2 Experimental design, materials and methods

The materials, the preparation of eGFP-His_6_ and GUVs, the optical setup used and the FCS data acquisition/analysis were described elsewhere [1].

### 2.1 Determination of average diffusion coefficients

**Fig. 1:**
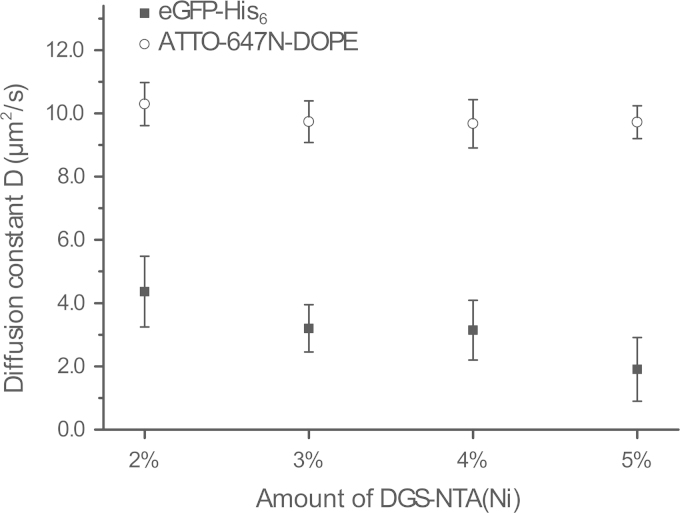
Diffusion coefficients determined by GUV-FCS assay. D for eGFP-His_6_ coordinated to NTA(Ni) (filled squares) and the membrane dye ATTO‐647N‐DOPE (circles) with increasing amounts of DGS-NTA(Ni). Error bars represent the combined standard error of mean. The D of ATTO‐647N‐DOPE shows no significant differences, whereas the D of eGFP-His_6_ decreases with increasing amounts of DGS-NTA(Ni).

We determined the average diffusion coefficients D of eGFP-His_6_ attached to DGS-NTA(Ni) in the lipid bilayer and of ATTO‐647N‐DOPE (Table 1 and Fig. 1) by applying the following equation:

(1) $$D=\frac{{\omega_{0}}^{2}}{4\tau_{2D}}$$

The average focal waist $w_{0}$ obtained from a calibration with Alexa488 and with ATTO-655, were $w_{0}=218.0\pm 6.0\;\mathrm{nm}$ (mean ± s.e.m, *n*=19) and $w_{0}=246.2\pm 4.6\;\mathrm{nm}$ (mean ± s.e.m, *n*=19), respectively. The diffusion times $\tau_{2D}$ were determined fitting the auto-correlation curves with a weighted 2*D*−3*D*+*T* model function. The D values were averaged and the significance of their deviation was tested using a one-way analysis of variance (ANOVA) in SigmaPlot 12.3 (Systat Software, Inc., San Jose, CA). This statistical analysis indicated a significance of deviation for the average diffusion coefficients of eGFP-His_6_ in presence of different DGS-NTA(Ni) concentrations (*F*(3,78)=19.48, *p*<0.001). With increasing amount of DGS-NTA(Ni), the eGFP-His_6_ average diffusion coefficients decreases from $D=4.36\pm 1.12\;µm^{2}/s$ (mean ± combined s.e.m., *n*=548) to $D=1.90\pm 1.01\;µm^{2}/s$ (mean ± combined s.e.m., *n*=593). In contrast, the average diffusion coefficient of ATTO-647N DOPE for all concentrations DGS-NTA(Ni) was $D=9.81\pm 0.70\;µm^{2}/s$ (mean ± combined s.e.m., *n*=3123) and did not show any statistical significant difference (*F*(3,86)=3.24, *p*=0.026).

### 2.2 *K*_*d*_ for eGFP-His_6_ DGS-NTA(Ni) system

Only in cases where the protein-lipid binding is purely stoichiometric and if the stoichiometry is known, the protein affinity for the lipid membrane can be expressed by the dissociation constant $K_{d}$. In equilibrium, an identical number of molecules P will dissociate from and associate to the lipid phase L per area and time P+nL→nPL. For 1:1 binding stoichiometry (n=1), $K_{d}$ is defined as:

(2) $$K_{d}=\frac{\left[P_{f}\right]\left[L_{f}\right]}{[\mathrm{PL}]}$$

where $\left[P_{f}\right]$ is the freely diffusing species in solution, $\left[\mathrm{PL}\right]=\left[P_{m}\right]$ the membrane associated fraction and $\left[L_{f}\right]=\left[L\right]-[L_{m}]$ with the total accessible lipid concentration $\left[L\right]\gg \left[L_{m}\right]$. Thus,

(3) $$K_{d}=\frac{\left[P_{f}\right]\left[L\right]}{[P_{m}]}=\frac{k_{\mathrm{off}}}{k_{\mathrm{on}}}$$

[L] is constant in a given sample and can be expressed by:

(4) $$\left[L\right]=\frac{A}{A_{L}N_{A}V}$$

Here, A is the total accessible lipid area, $A_{L}$ the area per lipid, $N_{A}$ the Avogadro׳s constant and V the volume of the sample chamber. $\left[P_{f}\right]$ and $\left[P_{m}\right]$ can be determined by FCS [1]. In particular, $\left[P_{m}\right]$ is obtained by:

(5) $$\left[P_{m}\right]=\left[P_{2D}\right]\frac{A}{V}$$

where $\left[P_{2D}\right]$ is the surface concentration on the top pole of a GUV.

A rearrangement of Eq. (3) gives:

(6) $$\left[P_{m}\right]=\frac{[L]}{K_{d}}\left[P_{f}\right]$$

Combining Eq. (6) with Eqs. (4) and (5) gives the following main equation (A and V cancel out):

(7) $$\left[P_{2D}\right]=\frac{1}{K_{d}A_{L}N_{A}}\left[P_{f}\right]$$

When a set of $\left[P_{f}\right]$ and $\left[P_{2D}\right]$ is plotted and fitted with a linear equation passing through the origin of the axis, $K_{d}$ can be calculated from the slope a:

(8) $$K_{d}=\frac{1}{aA_{L}N_{A}}$$

Comparing Eq. (8) with Eq. (7) in Thomas *et al.* [1] leads to the following conversion between $K_{d}$ and partition coefficient $K_{P}$:

(9) $$\frac{K_{P}}{W}=\frac{1}{K_{d}}$$

with the water concentration [W] being constant with $\left[W\right]=W=55.5\;M$.

**Fig. 2:**
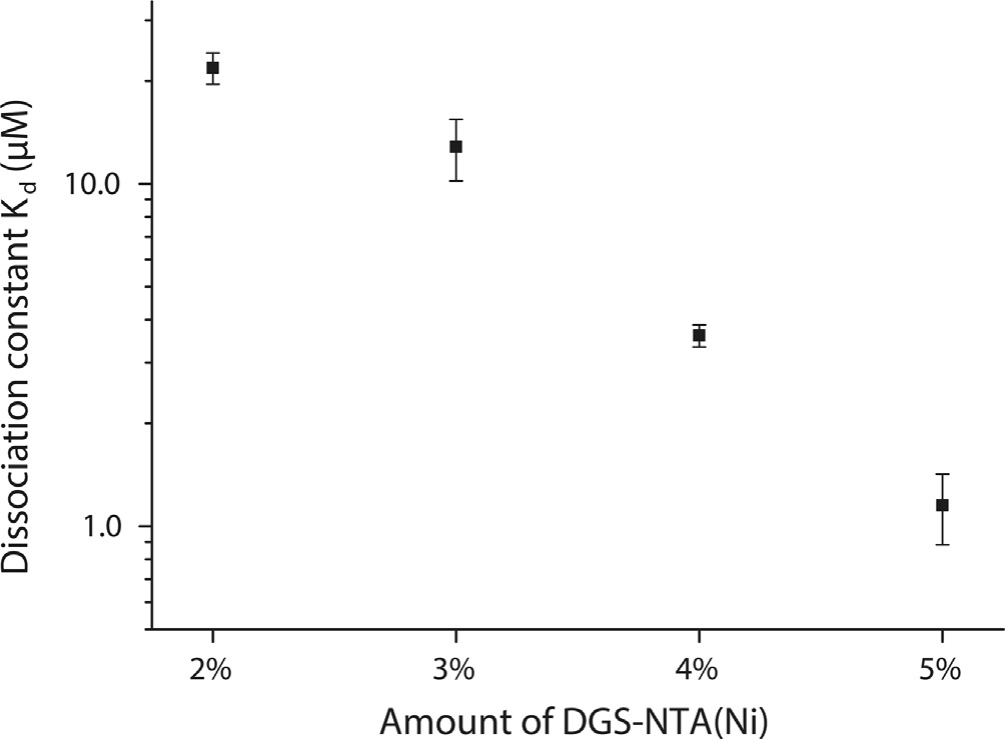
Graphic presentation of dissociation constant $K_{d}$ obtained by GUV-FCS assay. Error bars represent the combined standard error of mean.

Assuming that the binding stoichiometry for the NTA(Ni)/eGFP-His_6_ system is 1:1 [2], [7], we could calculate the dissociation constant $K_{d}$ from the reported partitioning coefficient $K_{P}$ [1] with Eq. (9) or directly from the slope a with Eq. (8). In Table 2 and Fig. 2 the values of the dissociation constant $K_{d}$ are given for the different content of DGS-NTA(Ni). They correspond to the upper range of values reported in the literature, which vary from 10 nM to 10 μM [7], [8], [9].

## Figures and Tables

**Table 1:** Diffusion coefficient D determined by GUV-FCS assay. Calculated diffusion coefficients by averaging all data points for increasing amounts of DGS-NTA(Ni) via the GUV method (mean ± combined s.e.m.).

**DGS-NTA(Ni)**	**eGFP-His**_**6**_ D **in µm**^**2**^**/s**	**ATTO‐647N‐DOPE D in µm**^**2**^**/s**
2%	4.36 ± 1.12 (*n*=548)	10.03 ± 0.68 (*n*=549)
3%	3.20 ± 0.75 (*n*=775)	9.74 ± 0.66 (*n*=900)
4%	3.14 ± 0.94 (*n*=740)	9.67 ± 0.76 (*n*=969)
5%	1.90 ± 1.01 (*n*=593)	9.72 ± 0.52 (*n*=705)


**Table 2:** $K_{d}$ determined by GUV-FCS assay. Calculated dissociation constants by fitting all data points for increasing amounts of DGS-NTA(Ni) via the GUV-FCS method (mean ± combined s.e.m.).

**DGS-NTA(Ni)**	$K_{d}$**in M**
2%	2.18 ± 0.23 · 10^−5^
3%	1.28 ± 0.26 · 10^−5^
4%	3.60 ± 0.27 · 10^−6^
5%	1.15 ± 0.27 · 10^−6^

